# A Facile Synthesis of ZnCo_2_O_4_ Nanocluster Particles and the Performance as Anode Materials for Lithium Ion Batteries

**DOI:** 10.1007/s40820-016-0122-4

**Published:** 2016-12-26

**Authors:** Yue Pan, Weijia Zeng, Lin Li, Yuzi Zhang, Yingnan Dong, Dianxue Cao, Guiling Wang, Brett L. Lucht, Ke Ye, Kui Cheng

**Affiliations:** 1 Key Laboratory of Superlight Materials and Surface Technology of Ministry of Education, College of Materials Science and Chemical Engineering, Harbin Engineering University, Harbin, 150001 People’s Republic of China; 2 Department of Chemistry, University of Rhode Island, Kingston, RI 02881 USA; 3 Department of Chemical Engineering, University of Rhode Island, Kingston, RI 02881 USA

**Keywords:** ZnCo_2_O_4_ nanocluster particles, Hydrothermal method, Sodium dodecyl benzene sulfonate, Lithium ion batteries

## Abstract

**Abstract:**

ZnCo_2_O_4_ nanocluster particles (NCPs) were prepared through a designed hydrothermal method, with the assistance of a surfactant, sodium dodecyl benzene sulfonate. The crystalline structure and surface morphology of ZnCo_2_O_4_ were investigated by XRD, XPS, SEM, TEM, and BET analyses. The results of SEM and TEM suggest a clear nanocluster particle structure of cubic ZnCo_2_O_4_ (~100 nm in diameter), which consists of aggregated primary nanoparticles (~10 nm in diameter), is achieved. The electrochemical behavior of synthesized ZnCo_2_O_4_ NCPs was investigated by galvanostatic discharge/charge measurements and cyclic voltammetry. The ZnCo_2_O_4_ NCPs exhibit a high reversible capacity of 700 mAh g^−1^ over 100 cycles under a current density of 100 mA g^−1^ with an excellent coulombic efficiency of 98.9% and a considerable cycling stability. This work demonstrates a facile technique designed to synthesize ZnCo_2_O_4_ NCPs which show great potential as anode materials for lithium ion batteries.

**Graphical Abstract:**

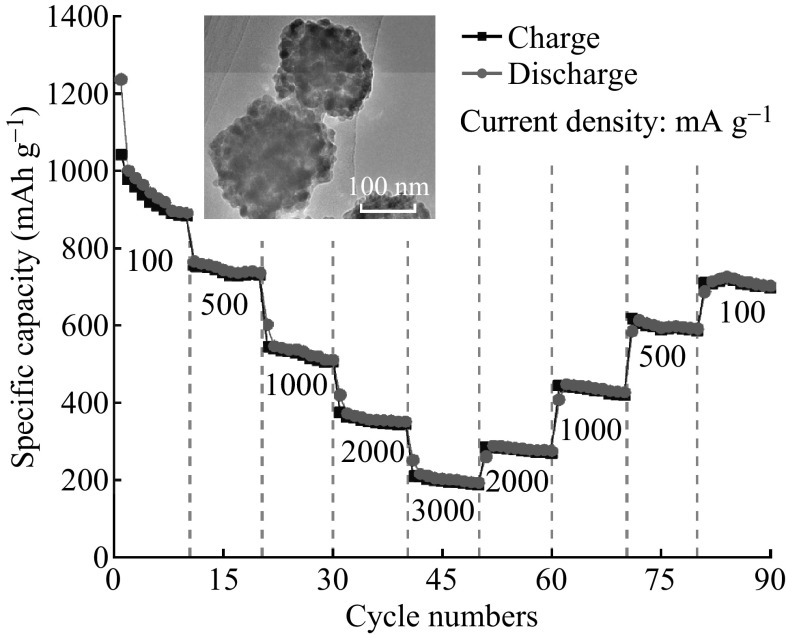

## Highlights

- ZnCo_2_O_4_ nanocluster particles (NCPs) were prepared through a hydrothermal method with the assistance of sodium dodecyl benzene sulfonate (SDBS).
- The ZnCo_2_O_4_ NCPs exhibit excellent rate performance. The initial lithiation-specific capacity of ZnCo_2_O_4_ NCPs with a current density of 100 mA g^−1^ reached 1110 mAh g^−1^ with a coulombic efficiency of 84.7 %, and a high delithiation capacity of 700 mAh g^−1^ was achieved over 100 cycles.

## Introduction

It is well known that novel renewable energy sources and energy storage materials are two major challenges in electrochemical technology. Rechargeable lithium ion batteries (LIBs), which have been recognized as vitally important devices of power sources, have attracted widespread attention. LIBs with high energy and power density, low cost, and short charging time are needed urgently to meet the rapid development of hybrid and electric vehicles. In principle, the electrochemical performance of safe LIBs depends largely on the electrode materials for lithium storage.

Among the array of promising anode materials for LIBs, transition metal oxides have been widely studied due to their higher specific capacities compared to traditional graphite with a specific capacity of 372 mAh g^−1^. Ternary oxides, AB_2_O_4_ (A=Mg, Mn, Fe, Co, Ni, Cu, or Zn; B=Mn, Fe, Co, Ni, or Cu; A≠B), with a variety of crystal structures (spinel, scheelite, brannerite, etc.) have been investigated as anode materials for LIBs [1–4]. This class of materials contains at least one transition metal ion and one or more electrochemically active/inactive ions. AB_2_O_4_ in previous electrochemical studies were synthesized via molten salt method [5–8], oxalate decomposition method [9, 10], combustion method [11, 12], solvothermal method [13], etc. And they were found to show good Li cyclability with relatively high specific capacities.

The typical ternary oxide, zinc cobaltite (ZnCo_2_O_4_), possesses a spinel structure, where the Zn^2+^ occupies the tetrahedral sites and the Co^3+^ occupies the octahedral sites. ZnCo_2_O_4_ has been demonstrated to be a promising candidate as anode materials for LIBs because of the outstanding electrochemical performance (the theoretical specific capacity of 975 mAh g^−1^) and the abundant source, low cost, and low toxicity of zinc. Generally, the electrochemical performance of electrode materials depends on the preparation technique, the size and shape of particles and the morphology. The strategies deployed to prepare ZnCo_2_O_4_ are similar to those designed to synthesize AB_2_O_4_ mentioned above [1, 14].

Hao [15] reported porous ZnCo_2_O_4_ microspheres synthesized by a solvothermal method, with a high reversible capacity of 940 mAh g^−1^ at 0.1 °C. In Huang’s work [16], core–shell ZnCo_2_O_4_ microspheres were fabricated by a hydrothermal method. They showed an initial discharge capacity of 1280 mAh g^−1^ at 200 mA g^−1^, and only 3.9% capacity was lost between the 2nd and the 5th cycles at 400 mA g^−1^. According to Zhao’s study [17], highly ordered mesoporous spinel ZnCo_2_O_4_ was prepared with SBA-15 as templates. It displayed a high reversible capacity of 1623 mAh g^−1^ at 2.0 A g^−1^. The capacity still remained at 1470 mAh g^−1^ with a high current density of 8.0 A g^−1^. Wang’s group [18] prepared hierarchical porous ZnCo_2_O_4_ microspheres by simply decomposing PBA followed by sintering at 550 °C, which showed an initial lithiation and delithiation capacity of 1737.1 and 1051.6 mAh g^−1^, respectively, after 100 cycles at 100 mA g^−1^. In general, nanosized ZnCo_2_O_4_ with uniquely designed structures showed promising results in enhancing the electrochemical performance due to the high surface-to-volume ratio and the excellent electronic transport property. However, the limitation for the industrial application of this anode material is the control in preparation of the active material.

Herein, a facile approach is designed to synthesize uniform ZnCo_2_O_4_ NCPs. The cycling stability study of our ZnCo_2_O_4_ NCPs shows a delithiation capacity of 700 mAh g^−1^ over 100 cycles under a current density of 100 mA g^−1^. Excellent electrochemical performance of ZnCo_2_O_4_ NCPs demonstrates that it is promising to employ this material in high-energy storage devices.

## Experiments

### Preparation of ZnCo_2_O_4_ NCPs and Structure Characterization

**Scheme 1:**
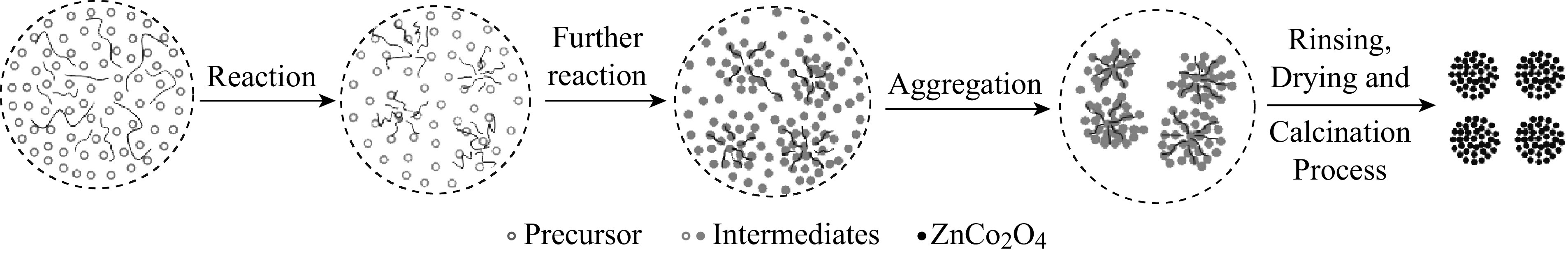
Illustration of the formation process of ZnCo_2_O_4_ NCPs

With the assistance of sodium dodecyl benzene sulfonate (SDBS), a non-aqueous hydrothermal method was designed for the synthesis of ZnCo_2_O_4_ NCPs. In a typical synthesis procedure, ZnCl_2_·H_2_O, CoCl_2_·H_2_O, urea, and SDBS were added into ethylene glycol. Afterwards, the mixture was stirred until the complete dissolution of all reagents occurred. After being transferred into a Teflon-lined autoclave, the pink and purple solution was subsequently kept constant at 200 °C for 24 h. After completely cooling down, the resulting pink precipitates were washed several times with a mixture of deionized water and absolute ethanol, and dried in a vacuum oven at 90 °C overnight. ZnCo_2_O_4_ NCPs were obtained by annealing the pink precipitates at 500 °C for 2 h in air. The hypothesized evolution of ZnCo_2_O_4_ NCPs is further illustrated in Scheme 1.

The morphology and structure of ZnCo_2_O_4_ NCPs were examined by a combination of scanning electron microscopy (SEM), transmission electron microscopy (TEM), and X-ray diffraction (XRD). Thermal analysis of the pink precursor power was characterized by thermogravimetry–differential thermal analysis (TG–DTA). The specific surface area of pure ZnCo_2_O_4_ NCPs powder was measured on Micromeritics Instrument Corporation TriStar II 3020 using N_2_ adsorption–desorption isotherms at −196 °C.

### Electrochemical Characterization

CR2032 coin cell was used to carry out the electrochemical experiments with Li foil serving as a reference and a counter electrode. Slurries of the active material (ZnCo_2_O_4_ NCPs), carbon black, and poly (vinyl difluoride) (PVDF; weight ratio of 70:20:10) in *N*-methyl-2-pyrrolidone were pasted on pure Cu foil with a thickness of 150 µm and dried under vacuum at 95 °C overnight to make working electrodes. The active material loading was 1.0–1.5 mg cm^−2^. 1.0 mol L^−1^ LiPF_6_ dissolved in a mixture of ethylene carbonate (EC), dimethyl carbonate (DMC), and ethyl methyl carbonate (EMC; volume ratio of 1:1:1) was used as the electrolyte. The cells were assembled in an Ar-filled glove box, with one microporous polypropene film (Celgard 2400) and one glass fiber as separator. An electrochemical workstation (VMP3/Z, Bio-logic, France) and a battery test system (CT-3008-5 V/5 mA, Neware Technology Ltd., Shenzhen, China) were used to test the electrochemical performance of all the cells under different current densities from 0.005 to 3.0 V vs. Li^+^/Li.

## Results and Discussion

### Structure and Morphology of ZnCo_2_O_4_

**Fig. 1:**
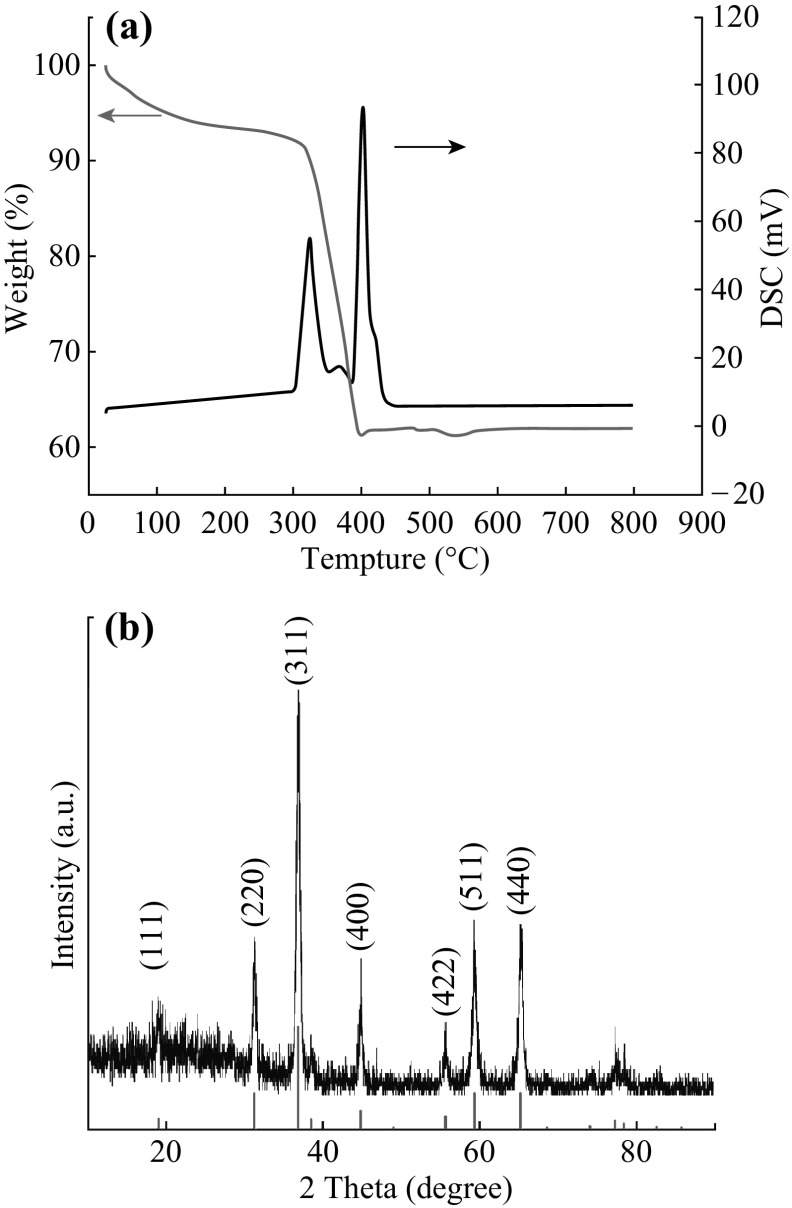
**a** TG–DTA of the precursor; **b** XRD of ZnCo_2_O_4_ NCPs

To determine a suitable calcination temperature to prepare ZnCo_2_O_4_ powder, TG–DTA was used and the result is shown in Fig. 1a. A small peak occurs at 367 °C, and two main exothermal peaks are at ~324 and ~402 °C, respectively. They are corresponding to the conversion of intermediates (metal glycoalates or alkoxides derivatives from the reaction of ethylene glycol with the metal ions) into ZnCo_2_O_4_ [19, 20]. Meanwhile, these peaks were accompanied by a drastic mass lose of about 29% in the temperature range 300–405 °C. In order to ensure that the precursor can be completely decomposed, the calcination temperature was finally set at 500 °C to prepare ZnCo_2_O_4_ NCPs.

Figure 1b shows the XRD pattern of synthesized ZnCo_2_O_4_ NCPs. The exhibited diffraction peaks can be indexed as a single cubic phase of ZnCo_2_O_4_ with the lattice constant a = 8.06 Å, in good agreement with the standard value of 8.09 Å (JCPDS card No. 23-1390). No peaks from other phases are detected, implying the high purity of synthesized ZnCo_2_O_4_ NCPs. Based on the Scherrer formula, the average diameter of ZnCo_2_O_4_ NCPs is around 13 nm calculated from the XRD pattern.

**Fig. 2:**
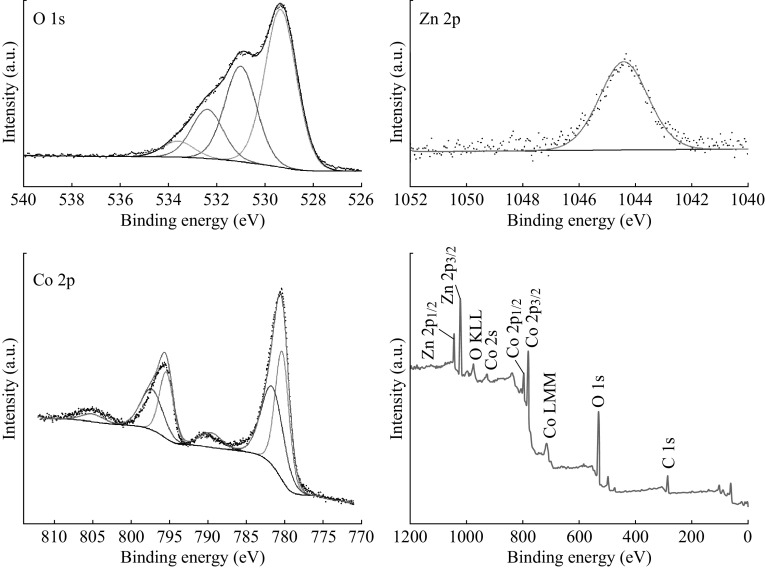
XPS spectra: O 1s, Zn 2p, Co 2p, and survey spectrum for the as-synthesized ZnCo_2_O_4_ NCPs

To investigate the composition and surface electronic state of ZnCo_2_O_4_ NCPs, XPS analysis was conducted. In the O 1s spectrum (Fig. 2), there are two main peaks at 529.2 and 530.8 eV, which should be attributed to the lattice oxygen from ZnCo_2_O_4_ NCPs and the oxygen from hydroxide ions, respectively. The two minor O 1s peaks around 532.2 and 533.7 eV are believed to be generated from surface bound water or adsorbed oxygen [21–23]. There are two major peaks at binding energies of 1044.4 and 1021.3 eV in the Zn 2p spectrum, attributed to Zn 2p1/2 and Zn 2p3/2 of Zn^2+^ [24]. The binding energy values of the two major peaks are 780.4 and 795.2 eV in the Co 2p spectrum, associated with Co 2p3/2 and Co 2p1/2, respectively. Additionally, the spineorbit splitting of the mentioned two peaks is 14.8 eV. Two accompanied weak satellite peaks are also visible at 790.0 and 805.0 eV and the energy gap between the main peak and the satellite peaks is around 9.8 eV. This suggests that Co cation can be assigned a value of +3 [15]. The results are quite close to those reported about MCo_2_O_4_ (M=Mg, Cu, Zn) [8]. In addition, the survey spectrum shows the presence of Zn, Co, and O as well as C.

**Fig. 3:**
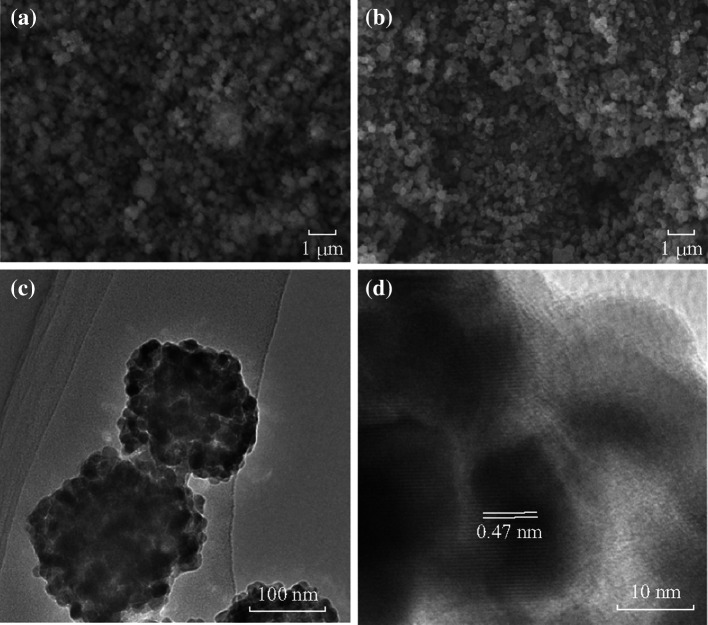
**a** SEM of the precursor. **b** SEM of ZnCo_2_O_4_ NCPs. **c** TEM of ZnCo_2_O_4_ NCPs. **d** HRTEM of ZnCo_2_O_4_ NCPs

The morphology of ZnCo_2_O_4_ NCPs was characterized by SEM and TEM, as shown in Fig. 3. The SEM images of the precursor and ZnCo_2_O_4_ are exhibited, respectively, in Fig. 3a, b. A uniform powder has been synthesized by the hydrothermal method with the assistance of SDBS. The sizes of precursor and ZnCo_2_O_4_ particles are approximately the same, and the fluffy surface turns to be tighter with the process of calcinations. From Fig. 3c, the ZnCo_2_O_4_ NCPs comprise small primary nanoparticles with a diameter around 10 nm. Figure 3d shows a typical HRTEM image of the ZnCo_2_O_4_ particles, revealing a structurally uniform lattice spacing of about 0.47 nm, which corresponds to the (111) lattice plane of the ZnCo_2_O_4_. Meanwhile, the crystallization of ZnCo_2_O_4_ is also well confirmed. The BET surface area of ZnCo_2_O_4_ NCPs is 30.0 m^2^ g^−1^, which is similar to those of other metal oxides or oxysalts as electrode materials for LIBs reported recently [25–31].

### Electrochemical Performance of ZnCo_2_O_4_ NCPs

**Fig. 4:**
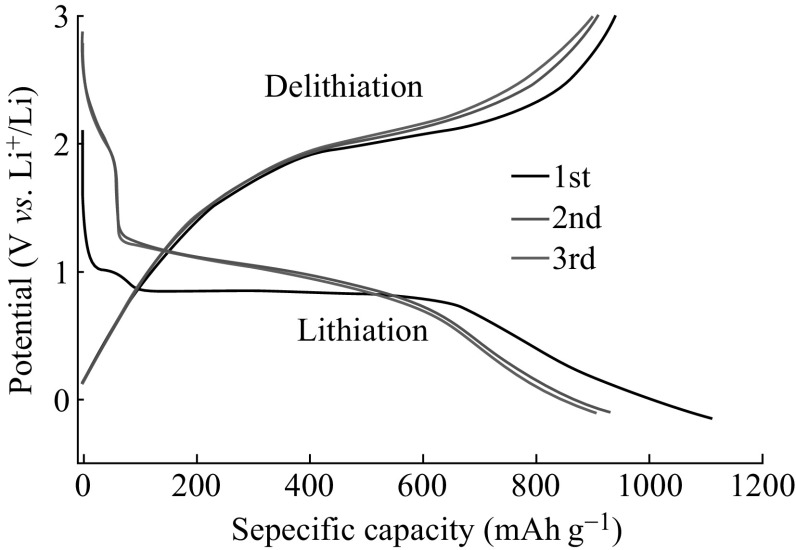
The first three lithiation/delithiation curves of ZnCo_2_O_4_ NCPs

The electrochemical performance of the ZnCo_2_O_4_ NCPs as the anode materials for LIBs was evaluated by galvanostatic discharge/charge experiments. The curves were measured at room temperature with a current density of 100 mA g^−1^ ranging from 0.005 to 3 V(vs. Li^+^/Li). Figure 4 shows the voltage-capacity profile of prepared ZnCo_2_O_4_ NCPs electrode for the first three lithiation/delithiation cycles. There is a stable potential plateau around 0.85 V during the first discharge process and the long discharging plateau becomes steeper and moves upward, consequently forming a long slope between 1.25 and 0.60 V in the following two cycles. The first lithiation capacity reaches as high as 1110(±5) mAh g^−1^ with a coulombic efficiency of 84.7% in the 1st cycle. The irreversible capacity may be attributed to the kinetic limitations of reactions [32], the formation of solid electrolyte interphase (SEI), the polymeric layer formation on the metal and nanoparticles (active material) under the deep discharge conditions (0.005 V vs. Li) [33], and the reduction of active metal to metal with Li_2_O formation, which is commonly observed for several types of electrode materials [34–36]. The lithiation and delithiation capacities in the 2nd cycle are 932 and 912(±5) mAh g^−1^, respectively. And the values change to 908 and 901(±5) mAh g^−1^, with a higher coulombic efficiency of 99.3% in the 3rd cycle. The capacities are continuously lost through the pulverization and aggregation of ZnCo_2_O_4_ NCPs, as well as the reduced electrical contact.

**Fig. 5:**
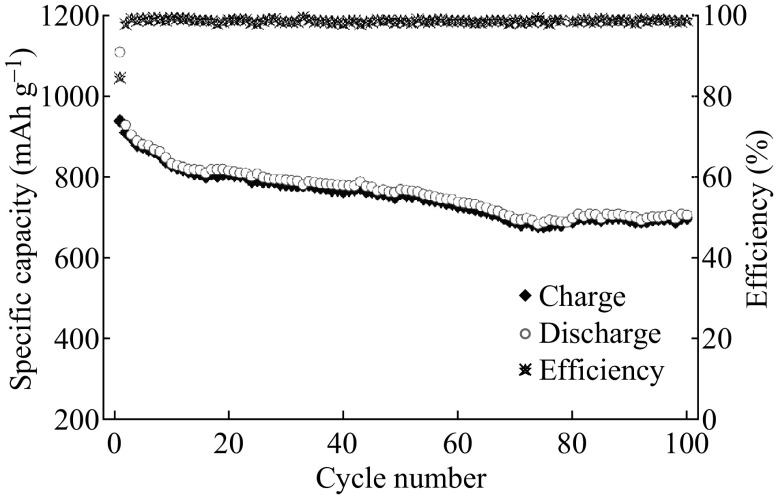
Cycling performance at 0.1 °C for ZnCo_2_O_4_ NCPs

The cycling stability and corresponding coulombic efficiency of ZnCo_2_O_4_ NCPs are demonstrated in Fig. 5. The ZnCo_2_O_4_ electrode reveals a large capacity fading during the initial 16 cycles. For the 16th cycle, the retention of delithiation capacity is 85.2%. In the following cycles, the reversible capacities decrease at a slower rate and a retention value about 81.0% is maintained in the 50th cycle. The decay rate of delithiation capacities increases until the 76th cycle, where it has a slight rebound with the continuous cycling. After 100 cycles, a high delithiation capacity of 700(±5) mAh g^−1^ is still retained with a retention of 74.4%, demonstrating the high specific capacity and superior cyclability of ZnCo_2_O_4_ NCPs. The coulombic efficiencies are ranging from 99.3% to 98.4% except in the first two cycles. The outstanding electrochemical behavior of ZnCo_2_O_4_ NCPs could be attributed to the unique structure (shown in Fig. 2c), which has possessed high specific surface area and empty space among the aggregated nanosized primary ZnCo_2_O_4_ particles. In this way, ZnCo_2_O_4_ NCPs can provide a short pathway for Li^+^ diffusion and a large electrode–electrolyte contact area for Li^+^ migration across the interface. More importantly, the empty space between adjacent particles can significantly improve the structural integrity caused by the volume change associated with the repeated lithiation and delithiation processes.

**Fig. 6:**
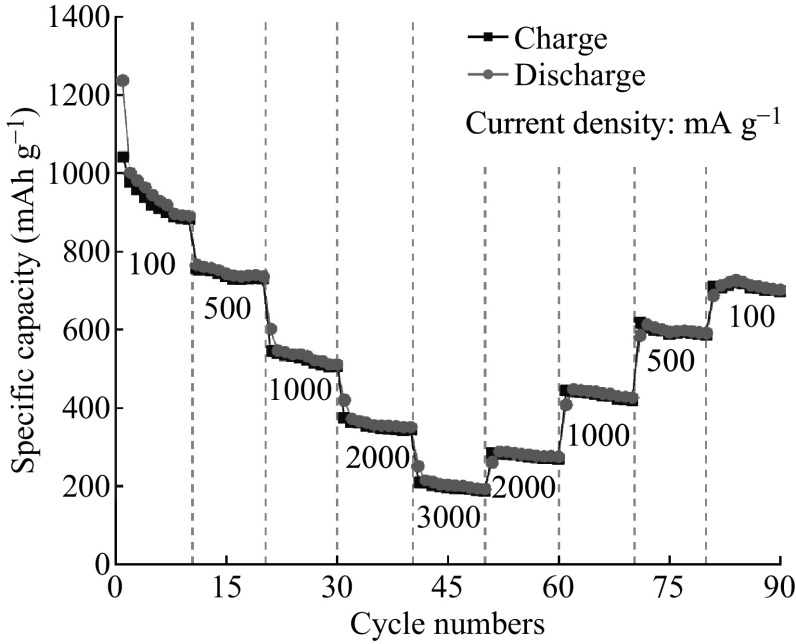
Rate performance of ZnCo_2_O_4_ NCPs electrode at various current densities

The rate performance of ZnCo_2_O_4_ NCP electrode is provided in Fig. 6. The reversible delithiation capacity is 884(±5) mAh g^−1^ at the 10th cycle under 100 mA g^−1^, and this value decreases to 731, 506, 345, 188(±5) mAh g^−1^ with a continuously increasing current density from 500 to 3000 mA g^−1^. More importantly, as the current density gradually decreases from 3000 back to 100 mA g^−1^, the ZnCo_2_O_4_ NCP electrode also shows good performance with slight decay. A reversible delithiation capacity of 698(±5) mAh g^−1^ could be resumed and maintained at the last cycle when the current is back to 100 mA g^−1^. This result demonstrates the excellent performance of ZnCo_2_O_4_ NCPs.

According to previous studies [14, 15, 37], the electrochemical reactions of ZnCo_2_O_4_ involved in the lithium insertion and extraction reactions can be illustrated as follows:

1 $${\text{ZnCo}}_{ 2} {\text{O}}_{ 4} + {\text{ 8Li}}^{ + } + {\text{ 8e}}^{ - } \to {\text{ Li}}_{x} {\text{ZnCo}}_{ 2} {\text{O}}_{ 4} \to {\text{ Zn}} + {\text{ 2Co }} + {\text{ 4Li}}_{ 2} {\text{O}}$$

2 $${\text{Zn }} + {\text{ Li}}^{ + } + {\text{ e}}^{ - } \leftrightarrow {\text{LiZn}}$$

3 $${\text{Zn }} + {\text{ Li}}_{ 2} {\text{O}} \leftrightarrow {\text{ ZnO }} + {\text{ 2Li}}^{ + } + {\text{ 2e}}^{ - }$$

4 $$2 {\text{Co }} + {\text{ 2Li}}_{ 2} {\text{O}} \leftrightarrow {\text{ 2CoO }} + {\text{ 4Li}}^{ + } + {\text{ 4e}}^{ - }$$

5 $$2 {\text{CoO }} + { 2}/ 3 {\text{Li}}_{ 2} {\text{O}} \leftrightarrow { 2}/ 3 {\text{Co}}_{ 3} {\text{O}}_{ 4} + { 4}/ 3 {\text{Li}}^{ + } + { 4}/ 3 {\text{e}}^{ - }$$

**Fig. 7:**
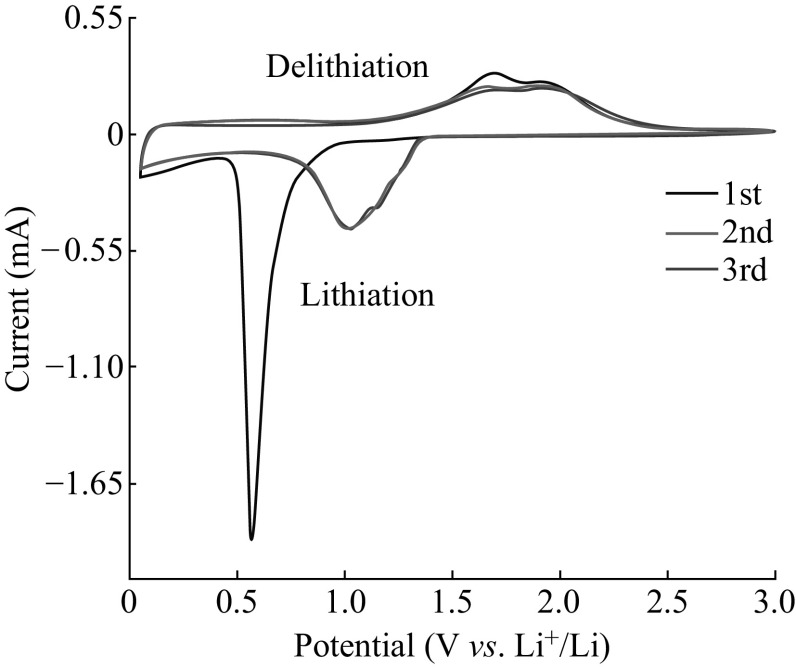
Cyclic Voltammograms of ZnCo_2_O_4_ NCPs electrode at a scan rate of 0.1 mV s^−1^

Cyclic voltammetry can provide additional detail on the electrochemical reactions of ZnCo_2_O_4_ NCPs with the electrolyte. Figure 7 presents the first three cyclic voltammograms of ZnCo_2_O_4_ NCPs electrode in the voltage of 0.005–3.0 V at a scan rate of 0.1 mV s^−1^. The initial cathodic process observed on the electrode starts at ~0.8 V and a sharp peak occurs at ~0.6 V versus Li, which should be resulted from the intercalation reaction of Li_*x*_ZnCo_2_O_4_, the reduction of Zn^2+^ and Co^3+^ to Zn^0^ and Co^0^ (Eq. 1), the formation of Li–Zn alloys (Eq. 2), and an irreversible reaction related to the decomposition of the electrolyte [14, 38]. In the anodic sweep, two main oxidation peaks are observed at 1.7 and 2.0 V characteristic of the oxidation process of Zn and Co to Zn^2+^ and Co^3+^ (Eqs. 3–5) [39]. The second CV scan contains a cathodic peak ~1.0 V, distinguishing the reduction mechanism from that in the 1st cycle [40] and two anodic peaks at 1.7 and 2.0 V. Similar CV scan is observed in the 3rd cycle although the intensity of all peaks decreases slightly, typical for reversible lithium ion intercalation/deintercalation and reversible cycling of the cells above.

**Fig. 8:**
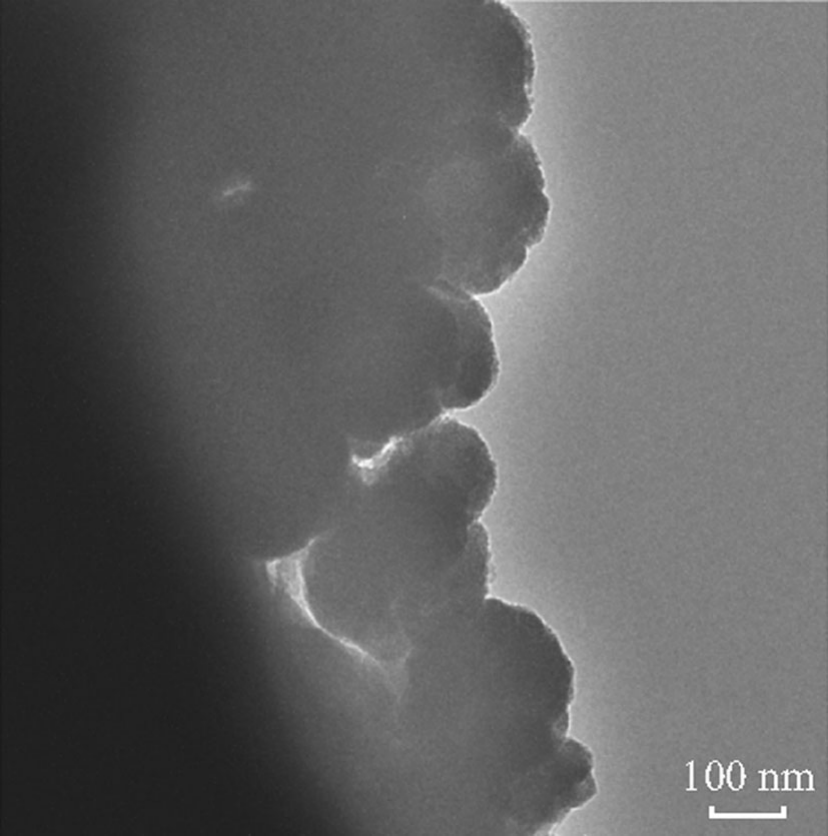
TEM image of ZnCo_2_O_4_ NCPs electrode after 100 cycles at 100 mA g^−1^

In order to investigate the morphology changes after continuous discharge and charge cycles, the cell of ZnCo_2_O_4_ NCPs after 100 cycles at 100 mA g^−1^ was disassembled and monitored by TEM. As revealed in Fig. 8, ZnCo_2_O_4_ NCPs anode after 100 cycles still shows well spherical morphology with a diameter ~ 100 nm. However, the primary clustered structure is not obvious after cycling test, which can be attributed to the irreversible structure destruction during the cycling process. In sum, these results strongly explain the reasons for the excellent electrochemical properties of ZnCo_2_O_4_ NCPs.

**Fig. 9:**
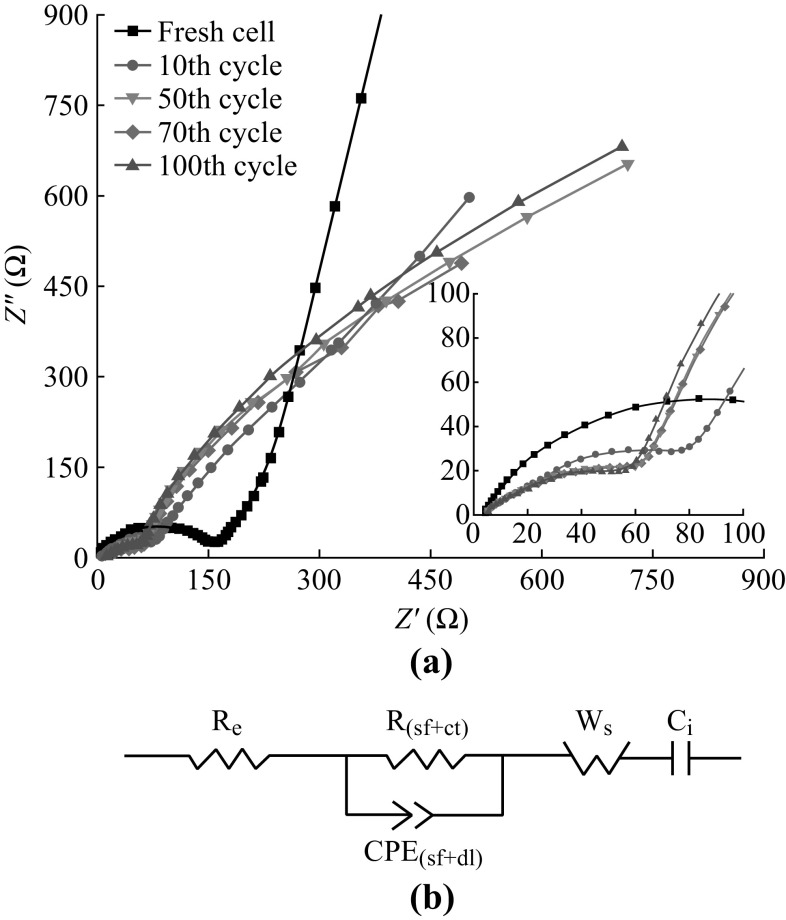
**a** Electrochemical impedance spectra of ZnCo_2_O_4_ NCPs electrode after different cycles. **b** Equivalent electrical circuit used to fit the data of Fig. 9a

Nyquist plots of ZnCo_2_O_4_ NCPs electrode at open-circuit voltage (OCV, 2.8–3.0 V) after different cycles at 0.1 °C are shown in Fig. 9a, to investigate how the impedance changes with continuous cycling. The impedance spectra were fitted to an equivalent circuit, consisting of the resistances for electrolyte, cell components, surface film (sf), and charge-transfer(ct); a constant phase element (CPE_*i*_); Warburg impedance (*W*_s_) and intercalation capacitance (*C*_*i*_) [32, 33]. The circuit is shown in Fig. 9b. The fitted impedance data values are listed in Table 1. The *R*_b_ values were relatively stable (~4.0 Ω) and *R*_(sf+ct)_ values were found to decrease with continuous cycle. The decrease of *R*_ct_ may be related to the wetting process between the ZnCo_2_O_4_ NCPs (active material) and electrolyte, as well as the lower polarization and higher reactivity of ZnCo_2_O_4_ NCPs. The CPE_(sf+ct)_ values increased from 12 µF (fresh cell) to 160 µF (after 10 cycles), corresponding to the formation of SEI film. After the 50th cycle, the values are almost stable. As cycling, the electrolyte can soak into the ZnCo_2_O_4_ particles, and the active ZnCo_2_O_4_ is converted to lower oxidation state, cobalt oxide, zinc oxide, and Li_2_O. This result is consistent with the cycling performance (Fig. 4).

**Table 1:** Impedance parameters of ZnCo_2_O_4_–Li after different cycles in the fully charged state

	Fresh cell	10th-charge cycle	50th-charge cycle	70th-charge cycle	100th-charge cycle
OCV (V vs. Li) open-circuit voltage	2.2	2.85	2.90	2.80	2.90
*R*_e_ (Ω) electrolyte resistance	3.9	4.4	4.0	4.2	4.1
*R*_(sf+ct)_ (Ω) surface film + charge transfer resistance	149.4	72.8	47.1	47.9	48.2
CPE_(sf+dl)_ (µF) constant phase element due to surface film + double layer capacitance	12	160	125	113	102
*W*_s_ (Ω) Warburg resistance	690	2969	3826	3838	3894
*C*_*i*_ (µF) intercalation capacitance	284	4.7	3.5	3.0	2.8

## Conclusions

In summary, ZnCo_2_O_4_ NCPs are synthesized successfully by a designed hydrothermal method with the assistance of SDBS. The characterizations by XRD, SEM, and TEM show uniform ZnCo_2_O_4_ NCPs around 100 nm in diameter, comprising aggregated primary ZnCo_2_O_4_ nanoparticles (~10 nm in diameter). The electrochemical measurements reveal that the first lithiation and delithiation capacities of ZnCo_2_O_4_ NCPs are 1110 and 941 mAh g^−1^, respectively. After 100 cycles, a high reversible delithiation capacity of 700 mAh g^−1^ is retained. The high capacities and good stability are attributed to the unique nanostructures of ZnCo_2_O_4_, which demonstrate the promising application of our synthesized ZnCo_2_O_4_ as anode materials for LIBs.

## References

[1] Reddy MV, Subba Rao GV, Chowdari BV (2013). Metal oxides and oxysalts as anode materials for Li ion batteries. Chem. Rev..

[2] Zhao D, Wang Y, Zhang Y (2011). High-performance Li-ion batteries and supercapacitors base on 1-D nanomaterials in prospect. Nano-Micro Lett..

[3] Pan Y, Zhang Y, Wei X, Yuan C, Yin J, Cao D, Wang G (2013). MgFe_2_O_4_ nanoparticles as anode materials for lithium-ion batteries. Electrochim. Acta.

[4] Xiao Y, Li X, Zai J, Wang K, Gong Y, Li B, Han Q, Qian X (2014). CoFe_2_O_4_-graphene nanocomposites synthesized through an ultrasonic method with enhanced performances as anode materials for Li-ion batteries. Nano-Micro Lett..

[5] Reddy MV, Yu C, Jiahuan F, Loh KP, Chowdari BVR (2012). Molten salt synthesis and energy storage studies on CuCo2O4 and CuO·Co3O4. RSC Adv..

[6] Reddy MV, Quan CY, Teo KW, Ho LJ, Chowdari BVR (2015). Mixed oxides, (Ni_1−*x*_Zn_*x*_)Fe_2_O_4_ (*x* = 0, 0.25, 0.5, 0.75, 1): molten salt synthesis, characterization and its lithium-storage performance for lithium ion batteries. J. Phys. Chem. C.

[7] Reddy MV, Rajesh M, Adams S, Chowdari BVR (2016). Effect of initial reactants and reaction temperature on molten salt synthesis of CuCo_2_O_4_ and its sustainable energy storage properties. ACS Sustain. Chem. Eng..

[8] Reddy MV, Xu Y, Rajarajan V, Ouyang T, Chowdari BVR (2015). Template free facile molten synthesis and energy storage studies on MCo_2_O_4_ (M=Mg, Mn) as anode for li-ion batteries. ACS Sustain. Chem. Eng..

[9] Darbar D, Reddy MV, Sundarrajan S, Pattabiraman R, Ramakrishna S, Chowdari BVR (2016). Anodic electrochemical performances of MgCo_2_O_4_ synthesized by oxalate decomposition method and electrospinning technique for Li-ion battery application. Mater. Res. Bull..

[10] Peshev P, Toshev A, Gyurov G (1989). Preparation of high-dispersity MCo_2_O_4_ (M=Mg, Ni, Zn) spinels by thermal dissociation of coprecipitated oxalates. Mater. Res. Bull..

[11] Cherian CT, Reddy MV, Rao GVS, Sow CH, Chowdari BVR (2012). Li-cycling properties of nano-crystalline (Ni_1−*x*_Zn_*x*_)Fe_2_O_4_ (0 ≤ *x* ≤ 1). J. Solid State Electrochem..

[12] Sharma Y, Sharma N, Rao GVS, Chowdari BVR (2007). Lithium recycling behaviour of nano-phase-CuCo_2_O_4_ as anode for lithium-ion batteries. J. Power Sources.

[13] Zhao H, Liu L, Xiao X, Hu Z, Han S, Liu Y, Chen D, Liu X (2015). The effects of Co doping on the crystal structure and electrochemical performance of Mg(Mn_2−*x*_Co_*x*_)O_4_ negative materials for lithium ion battery. Solid State Sci..

[14] Reddy MV, Kenrick KYH, Wei TY, Chong GY, Leong GH, Chowdari BVR (2011). Nano-ZnCo_2_O_4_ material preparation by molten salt method and its electrochemical properties for lithium batteries. J. Electrochem. Soc..

[15] Hao S, Zhang B, Ball S, Copley M, Xu Z, Srinivasan M, Zhou K, Mhaisalkar S, Huang Y (2015). Synthesis of multimodal porous ZnCo_2_O_4_ and its electrochemical properties as an anode material for lithium ion batteries. J. Power Sources.

[16] Huang L, Waller GH, Ding Y, Chen D, Ding D, Xi P, Wang ZL, Liu M (2015). Controllable interior structure of ZnCo_2_O_4_ microspheres for high-performance lithium-ion batteries. Nano Energy.

[17] Zhao R, Li Q, Wang C, Yin L (2016). Highly ordered mesoporous spinel ZnCo_2_O_4_ as a high-performance anode material for lithium-ion batteries. Electrochim. Acta.

[18] Wang D, Qi X, Gao H, Yu J, Zhao Y, Zhou G, Li G (2016). Fabricating hierarchical porous ZnCo_2_O_4_ microspheres as high-performance anode material for lithium-ion batteries. Mater. Lett..

[19] Sharma Y, Sharma N, Subba GV (2007). Rao, B.V.R. Chowdari, Nanophase ZnCo_2_O_4_ as a high performance anode material for Li-ion batteries. Adv. Funct. Mater..

[20] Deng Y, Zhang Q, Tang S, Zhang L, Deng S, Shi Z, Chen G (2011). One-pot synthesis of ZnFe_2_O_4_/C hollow spheres as superior anode materials for lithium ion batteries. Chem. Commun..

[21] Marco JF, Gancedo JR, Gracia M, Gautier JL, Ríos E, Berry FJ (2000). Characterization of the nickel cobaltite, NiCo_2_O_4_, prepared by several methods: an XRD, XANES, EXAFS, and XPS study. J. Solid State Chem..

[22] Jiménez VM, Fernández A, Espinós JP, González-Elipe AR (1995). The state of the oxygen at the surface of polycrystalline cobalt oxide. J. Electron Spectrosc. Relat. Phenom..

[23] Choudhury T, Saied SO, Sullivan JL, Abbot AM (1989). Reduction of oxides of iron, cobalt, titanium and niobium by low-energy ion bombardment. J. Phys. D.

[24] Wen XL, Chen Z, Liu Z, Lin X (2015). Structural and magnetic characterization of ZnCo_2_O_4_ thin film prepared by pulsed laser deposition. Appl. Surf. Sci..

[25] Kumar A, Jayakumar OD, Naik VM, Nazri GA, Naik R (2016). Improved electrochemical properties of solvothermally synthesized Li_2_FeSiO_4_/C nanocomposites: a comparison between solvothermal and sol-gel methods. Solid State Ion.

[26] Wang X, Liu Y, Arandiyan H, Yang H, Bai L, Mujtaba J, Wang Q, Liu S, Sun H (2016). Uniform Fe_3_O_4_ microflowers hierarchical structures assembled with porous nanoplates as superior anode materials for lithium-ion batteries. Appl. Surf. Sci..

[27] Zhang Y, Huang J, Ding Y (2016). Porous Co_3_O_4_/CuO hollow polyhedral nanocages derived from metal-organic frameworks with heterojunctions as efficient photocatalytic water oxidation catalysts. Appl. Catal. B.

[28] Nilmoung S, Sinprachim T, Kotutha I, Kidkhunthod P, Yimnirun R, Rujirawat S, Maensiri S (2016). Electrospun carbon/CuFe_2_O_4_ composite nanofibers with improved electrochemical energy storage performance. J. Alloys Compd..

[29] Narsimulu D, Rao BN, Venkateswarlu M, Srinadhu ES, Satyanarayana N (2016). Electrical and electrochemical studies of nanocrystalline mesoporous MgFe_2_O_4_ as anode material for lithium battery applications. Ceram. Int..

[30] Qin Y, Long M, Tan B, Zhou B (2014). RhB adsorption performance of magnetic adsorbent Fe_3_O_4_/RGO composite and its regeneration through a fenton-like reaction. Nano-Micro Lett..

[31] Pan Y, Ye K, Cao D, Li Y, Dong Y, Niu T, Zeng W, Wang G (2014). Nitrogen-doped graphene oxide/cupric oxide as an anode material for lithium ion batteries. RSC Adv..

[32] Cherian CT, Zheng M, Reddy MV, Chowdari BV, Sow CH (2013). Zn_2_SnO_4_ nanowires versus nanoplates: electrochemical performance and morphological evolution during Li-cycling. ACS Appl. Mater. Inter..

[33] Reddy MV, Subba Rao GV, Chowdari BVR (2011). Nano-(V_1/2_Sb_1/2_Sn)O_4_: a high capacity, high rate anode material for Li-ion batteries. J. Mater. Chem..

[34] Xiao C, Du N, Zhang H, Yang D (2014). Improved cyclic stability of Mg_2_Si by direct carbon coating as anode materials for lithium-ion batteries. J. Alloys Compd..

[35] Xu S, Lu L, Zhang Q, Jiang Q, Luo Z, Wang S, Li G, Feng C (2016). A facile synthesis of flower-like CuO as anode materials for lithium (sodium) ion battery applications. J. Nanosci. Nanotechnol..

[36] Liu HW, Liu HF (2016). Preparing micro/nano dumbbell-shaped CeO_2_ for high performance electrode materials. J. Alloys Compd..

[37] Zhong X-B, Wang H-Y, Yang Z-Z, Jin B, Jiang Q-C (2015). Facile synthesis of mesoporous ZnCo_2_O_4_ coated with polypyrrole as an anode material for lithium-ion batteries. J. Power Sources.

[38] Nie M, Chalasani D, Abraham DP, Chen Y, Bose A, Lucht BL (2013). Lithium ion battery graphite solid electrolyte interphase revealed by microscopy and spectroscopy. J. Phys. Chem. C.

[39] Li J, Wang J, Wexler D, Shi D, Liang J, Liu H, Xiong S, Qian Y (2013). Simple synthesis of yolk-shelled ZnCo_2_O_4_ microspheres towards enhancing the electrochemical performance of lithium-ion batteries in conjunction with a sodium carboxymethyl cellulose binder. J. Mater. Chem. A.

[40] Luo W, Hu X, Sun Y, Huang Y (2012). Electrospun porous ZnCo_2_O_4_ nanotubes as a high-performance anode material for lithium-ion batteries. J. Mater. Chem..

